# Supplementary material for: Are carbon emissions trading and green financial instruments synergistic? -Comprehensive quantitative research based on content analysis
Source: PLoS One. 2024 Mar 7;19(3):e0298601. doi: 10.1371/journal.pone.0298601 (PMC10919601; doi:10.1371/journal.pone.0298601)
Supplement: S1 Data — (ZIP) [file pone.0298601.s001.zip › Data/地级市Z_F工作报告.zip!/╔╜╬≈/╨├╓▌╩╨/└┤╫╘╣1⁄2╓┌║┼í╛┬φ┐╦╩2╛▌═°í┐_2013.docx]

各位代表：

　　现在，我代表市人民政府向大会报告工作，请予审议，并请市政协委员和其他列席会议人员提出意见。

　　一、2012年工作回顾

　　2012年是极不平凡的一年。市政府在省委、省政府和市委的正确领导下，认真贯彻落实党的十八大精神，全面实施“3581”发展战略，紧扣主题主线，坚持稳中求进，力争好中求快，深入开展“项目落地年”、“大干城建年”、“走出去年”活动，努力克服经济下行的压力，扎实推进各项工作，较好完成了年初确定的各项任务。

　　（一）经济保持平稳增长。认真落实中央和省委、省政府稳增长政策，制定促进增长的35条措施，出台加快服务业发展10项政策和加快发展民营经济36条意见。加强对经济发展趋势的研判，及时解决经济运行中的突出问题，千方百计促进经济稳定增长。全市生产总值完成620.9亿元，同比增长11.5%；固定资产投资完成653.3亿元，同比增长26.6%；规模以上工业增加值完成301.6亿元，同比增长16.4%；社会消费品零售总额完成215.5亿元，同比增长16.2%；财政总收入完成144.2亿元，同比增长18%；一般预算收入完成65.2亿元，同比增长21.6%；外贸进出口总额21800万美元，同比增长15.2%；城镇居民人均可支配收入18318元，同比增长15%；农民人均纯收入4776元，同比增长15.5%。多项经济指标增幅高于全省平均水平，各项约束性指标年度任务全部完成。我市被评为全省目标责任考核优秀市。

　　（二）项目攻坚收效显著。狠抓重点工程，推动投资增长。省、市重点工程投资完成854.76亿元，投资完成率111.4%；重点项目落地657项，落地金额1384.5亿元，同比增长1倍多；中博会、首届晋商大会等13次招商活动签约项目339个，投资额3190亿元，到位资金378亿元，同比增长80%。狠抓重点产业，夯实发展基础。兼并重组煤矿标准化建设顺利推进；风电装机突破100万千瓦；静乐“1830”、河曲同德化工项目竣工投产；定襄昊坤不锈钢等装备制造企业改造升级步伐加快；代县全向实业等农业产业化龙头企业发展壮大，百万元以上龙头企业销售收入达44亿元；五台山景区提升改造工程取得阶段性成果，芦芽山景区汾源公园全面开工，原平天涯山生态修复治理和宁武恢河治理进展顺利；忻州瑞新物流中心等一批现代商贸物流项目获批，煤炭物流业成为神池、五寨、偏关、岢岚等县财政增收、经济发展的重要支撑；涉及7大产业的70个转型发展重点项目有40个竣工投产、20个正在建设、10个开展前期工作，全市工业新兴产业投资增幅28%以上。攻坚重大项目，加快核准进度。河曲电厂二期2×660MW、神华河曲一期2×300MW项目获得核准，忻州广宇电厂二期热电联产、繁峙华茂二期、代州钢铁等项目获批。实施县（市、区）长联系“一园六企”制度。建设“一园”11个，入驻项目39个；建设“六企”84个，建成19个、在建48个、开展前期的17个。原平煤化工循环经济园区列入省级新型工业化示范基地培育园区。充分发挥观摩项目示范带动作用，迅速落实全省重点工作忻州观摩检查点评意见，努力推动项目建设向更高层次发展。

　　（三）农民脱贫增收势头较好。实施“两增三建”工程，推动“三农”更好发展。建设219万亩杂粮核心产业区，新增设施农业面积1.46万亩。全市粮食总产达到32.68亿斤，再创历史新高。引进江苏雨润等一批大型企业，全市农产品加工转化率达到48%。发展“一村一品”专业村275个，新认证“三品一标”产品88个，忻府区糯玉米获国家地理标志认证。加强农业基础建设，实施8大水利工程，河曲引黄灌溉工程通水到县城试验成功。500个新农村建设重点推进村和12个新农村连片区全部完成村级规划和“四化四改”、“五个一工程”建设任务。扎实推进扶贫攻坚。当年减贫8.5万人。贫困县农民人均纯收入4264元，同比增长22%。事关3.2万人的扶贫移民搬迁工程全部开工，共建房8629套，超过省里70%进度要求12.8个百分点。

　　（四）城镇化建设取得重大进展。抢抓城镇化历史机遇，认真落实“大干城建年”各项任务。中心城市实施“7451”城建重点工程，“七路”、“三桥”全部竣工通车，“五馆一院”奠基开工，火车站广场改造工程完工，城中村改造工作启动。13个县（市）城镇扩容提质明显加速，建成了一批各具特色的亮点工程。繁峙砂河、宁武阳方口等13个全省百镇建设重点镇完成年度计划投资的5倍。加强规划和建设管理，编制《忻州市城市总体规划》、《忻定原组群规划》和《市区扩容提质实施方案》，建成忻州市数字化城管系统。大力支持依法建设，及时查处违法建设，深入整顿土地建筑市场秩序。建立了防范违法建设新机制，形成了支持依法建设的浓厚氛围。成功敲响城区国有土地依法拍卖第一槌。

　　（五）生态环境持续改善。强化节能管理。开展对列入全省“千家企业”的61户企业和全市105户重点企业的节能目标考核评价，淘汰13户企业145万吨落后产能和4台2.4万千瓦小火电机组，实施40个重点节能改造项目。全面治理工业源、农业源、生活源污染，严格控制重点区域和主要流域的污染排放。15座污水处理厂正常运行，五台山污水处理厂按照世界遗产地标准升级扩建，五台、静乐、神池、五寨、岢岚、偏关6县垃圾处理场全部完工。实施农村连片整治示范工程，成功创建3个省级生态乡（镇）、25个省级生态村。19个县级以上集中饮用水源保护地水质达标率全部稳定在100%，提前5个月实现消灭劣五类水目标。积极推进中心城市创建全国环保模范城市、省级园林城市、国家可再生能源应用示范城市“三城同建”工作，城区绿化覆盖率提高5个百分点，建筑节能综合排名全省第一。推动县（市）创建山西省环保模范城市。神池县通过验收，保德等8个县“创模”工作扎实推进。全市14个县（市、区）及五台山风景区二级以上天数总计达到5455天，其中忻州城区达到356天。积极开展国家卫生城镇创建活动，定襄县城、河曲县城和忻府区奇村镇、宁武县东寨镇通过国家验收。狠抓造林绿化。营造林面积68.67万亩，超全年计划任务4.64万亩。认真实施大运高速、忻阜高速通道绿化工程，建立健全了有效的林木管护新机制。

　　（六）民生和社会事业全面发展。坚持把安全生产作为最大的民生工程，一以贯之，常抓不懈。实行安全隐患举报重奖和安全隐患事故重罚制度，狠抓安全生产培训和安全事故警示教育，针对重点领域和薄弱环节出台了一系列规定，把安全工作的触角向一线延伸，把安全工作的责任向基层传递，从源头上防范事故，努力推动本质安全。全年安全生产目标任务圆满完成，安全生产形势继续稳定好转，连续第三年位居全省先进行列。公路治超工作实现全省五连冠。全面完成农村新的“五个全覆盖”，制定下发9个具体管理办法，确保两轮“五个全覆盖”成果发挥长效作用。理顺城区供热体制，确保供热企业正常供暖、居民正常采暖。完成低收入农户1吨煤发放工作。为444个村建设饮水安全工程455处，解决了21.28万农村人口的饮水安全问题。开工建设各类保障性住房19836套、竣工2887套，出台10个规范性文件，确保保障性住房建设、分配始终在阳光下进行。全面完成6963户农村危房改造年度任务。加强社会保障和就业工作。认真落实城镇企业退休人员养老金增长政策，继续提高城市低保、农村低保和农村五保保障标准。城镇登记失业率控制在3.27%。实施农村义务教育学生营养改善工程，惠及6县680所学校79500名学生。强化科技支撑。全年专利申请739件，争取国家级科技项目4项、省级科技项目56项。狠抓医改重点工作。巩固基本药物制度和基层医药卫生体制综合改革成果，启动实施计生家庭奖励制度，优生优育水平不断提高。巩固文化体制改革成果。出台《支持改制后国有文艺院团发展的意见》，推进“十大文化工程”，我市被评为全国文化体制改革工作先进市。实施文化惠民工程。启动“周末大戏台”和“乡村大舞台”，举办忻州市第二届“梨花奖”舞台艺术大赛，为全市农村放映公益电影6万余场。设立政府重点文艺作品扶持资金，扶持市、县两级文化产业开发“十项基础行动”。《情悟五台山》摘取第二届全国戏剧文化奖调演7个奖项，《徐向前返晋》、二人台省城大拜年汇演引起强烈反响，北京“陈巨锁墨迹展”、“洛阳·忻州书画联展”获得圆满成功。文化基础设施建设力度加大，县级“三馆一院”纳入规划，一些地方开始启动实施。加快广电网络整合。山西广电信息网络集团忻州分公司挂牌，市广电融入全省网络，县级广电网络整合有序推进。全民健身活动蓬勃开展。成功举办第六届中国忻州摔跤节。

　　（七）改革开放不断深化。推进转型综改试验。确立建设“三个门户”、打造“三个集散地”、发展“六大经济板块”的转型综改重点，出台7个体制机制创新文件，全面实施转型综改2012年行动方案，加快“一市两县”、“一市两园”、“一县一企”试点建设，支持省级试点县原平市、市级试点县宁武县先行先试。攻坚市直国企改革。忻州钨丝厂转让国有产权、实现有序退出完成实质性步骤；忻通公司成功实现与阳煤集团重组，阳煤忻通公司正式挂牌；云河集团实行先立后破、减员消债、盘活资产，分立破产有序推进；市水务集团与省水投公司合作稳步推进。深化行政审批制度改革，市级审批事项由209项减少到109项。推进事业单位人事制度改革，除政策性安置外全部实现公开招聘。全力支持原平、保德扩权强县试点改革，该下放的权力全部下放。创新融资模式，全面推广“助保贷”项目，金融生态环境进一步优化。

　　（八）政府自身建设进一步加强。积极推动法治政府、为民政府、服务政府、效能政府、诚信政府、廉洁政府建设。认真执行市人大及其常委会的各项决议，主动接受市人大的法律监督、工作监督和市政协的民主监督。坚持向人大报告工作制度和向政协通报工作制度，积极支持市政协参政议政。办理人大代表建议107件、政协委员提案204件，办复率均达100%。重大事项坚持广泛征询民主党派、无党派代表人士以及群众团体和社团组织的意见，努力做到民主科学集体决策。深入开展“六五”普法，全面加强政府法制工作。坚持人民主体地位，开展“给人民汇报、向人民问计、请人民评议”活动。向全市政府系统工作人员提出“五章约法”，坚持言必行、行必果。深化政务公开，让权力在阳光下运行。坚持勤俭办事，反对铺张浪费，实行财政资金使用效益评价制度。狠抓政府系统廉政建设，严肃查处各类违法违纪案件。深入开展市场秩序整顿，发展环境进一步优化。加强和创新社会管理，认真化解社会矛盾，积极推进社区网格化管理。全面贯彻民族宗教政策，依法管理民族宗教事务。不断巩固军政军民团结，深入开展国防教育，切实加强预备役建设和国防后备力量建设。外事、侨务、统计、应急、人防、气象、地震、文物、档案等工作得到新加强，老龄、残疾人、红十字会等工作取得新进步。

　　各位代表！过去一年取得的成绩，是市委统揽全局、坚强领导的结果，是市人大、市政协大力支持、有力监督的结果，是全市上下团结奋斗、扎实工作的结果。在此，我代表市人民政府，向全市干部群众，向驻忻部队指战员、武警官兵和公安民警，向各民主党派、人民团体，向各驻忻单位，向所有关心支持忻州发展进步的朋友们，表示衷心的感谢！

　　我们清醒地认识到，目前我市经济社会发展还存在不少问题。全市人均GDP与全省相差1万多元，发展不足仍然是首要问题，必须做大经济总量，加速赶队前行。传统产业层次不高，新型产业发展缓慢，转变经济发展方式任务繁重，必须加快产业优化升级，稳步提升经济增长的质量和效益。新的经济增长点不多，重点项目推进不快，财政收支压力加大，必须牢固树立“产业第一”的观点，着力服务现有企业，营造重商、亲商、安商的浓厚氛围。新型农业生产经营主体发展滞后，农业基础仍然薄弱，农村扶贫开发压力较大，农民稳定增收任务艰巨，必须毫不动摇地抓好“三农”工作。社会管理和社区建设滞后，民生事业与群众期待有不小距离，必须把保障和改善民生作为全部工作的出发点和落脚点。环境保护和生态文明建设基础脆弱，安全生产和节能减排压力加大，必须在建设资源节约型、环境友好型、本质安全型社会上付出更大努力。城镇化建设刚刚起步，服务依法建设效率不高，违法建设仍然突出，土地节约集约利用程度不高，农村转移人口市民化政策措施准备不足，必须维护规划权威，严格依法办事，强化精细管理，稳定提高城镇化发展质量，推动城镇化健康发展。主动创新意识不强，攻坚克难办法不多，财政依赖、资源依赖、贫困依赖不同程度存在，必须解放思想、转变职能，深化改革、创优环境，立足自身、破题前行。

　　我们要通过扎实苦干和艰苦努力，坚持不懈地解决这些问题，加快推动忻州由工业小市向工业大市的跨越，由农业大市向农业强市的跨越，由文化旅游资源富市向文化旅游产业强市的跨越，由传统落后城市向现代宜居城市的跨越，加快建设富裕文明、开放和谐、充满活力的新型工业旅游城市，把全市人民建设美好家园、过上美好生活的殷切希望变成现实！

　　二、2013年工作安排

　　今年是全面贯彻落实十八大精神的开局之年，是实施“十二五”规划承前启后的关键一年，是为全面建成小康社会奠定坚实基础的重要一年。做好今年的政府工作，必须坚持以中国特色社会主义理论体系为指导，坚持主题主线，坚持稳中求进，抓住转型综改试验区建设重大历史机遇，认真实施“3581”发展战略，更加重视项目建设和新型产业体系培育，更加重视民主法治和改革开放，更加重视统筹城乡和民生改善，更加重视环境保护和生态文明建设，更加重视文化引领和软实力提升，认真开展“项目推进年”和第二个“大干城建年”活动，努力完成科学发展、转型跨越、赶队前行、进位争先的年度目标任务，加快建设富裕文明、开放和谐、充满活力的新型工业旅游城市，为忻州与全省同步建成全面小康社会而努力奋斗。

　　主要预期指标是：地区生产总值增长11%，固定资产投资增长23%，社会消费品零售总额增长15%，进出口总额增长6%，财政总收入增长13%，一般预算收入增长13%，城镇居民人均可支配收入增长13%，农民人均纯收入增长14%，城镇新增就业岗位3.3万个，城镇登记失业率控制在3.4%以内，居民消费价格总水平涨幅控制在3.5%左右。

　　约束性指标是：万元生产总值综合能耗下降3.5%，万元生产总值二氧化碳排放量下降3.7%，万元工业增加值用水量下降5.2%，二氧化硫、化学需氧量、氨氮、氮氧化物、烟尘、粉尘排放量完成省下达任务。

　　今年重点做好8个方面工作：

　　（一）增强经济发展动力，促进经济持续健康发展。

　　加大投资力度。抓好重点工程。今年安排省、市重点工程465项，总投资3928亿元，年度计划完成720亿元。其中，省重点工程74项，年度计划完成319亿元；市重点工程391项，年度计划完成401亿元。抓好重大基础设施项目。继续推进灵河高速等在建项目，抓紧实施五台山机场、机场连接线、108国道城区过境改线、集中连片特困地区公路改造升级等道路建设项目；推进山西大水网忻州项目区、南云中河治理、云中水厂建设、农村饮水安全、病险水闸除险加固等水利工程项目。抓好产业项目。实施产业项目292个，其中一产64个、二产170个、三产58个，总投资1615.2亿元，年度计划完成347.6亿元，进一步增强发展的后劲。

　　大力开展“项目推进年”活动。强化推进机制。实行储备、签约、落地、开工、建设、投产、服务、考核“八位一体”，进一步打开思路、坚定信心，咬定目标、明确责任，创优环境、招商引资，加快核准、及早开工，强力推进、搞好服务，切实加快项目建设。做实项目储备。今年招商引资签约250个项目、总投资2500亿元，储备1000个项目、总投资1万亿元。创新招商方式。向相关要素招商，向提升、延伸、转型三方面寻找合作伙伴，认真搞好大型活动走出去招商，深入开展以商招商、以企招商，积极探索市场化招商，确保招商引资任务的完成。

　　扩大居民消费。在提高消费能力、稳定消费预期、增强消费意愿、改善消费环境上下功夫，不断提高消费对经济增长的拉动力。积极发展电子商务、连锁经营等新型业态，大力倡导节能环保等绿色消费，有力促进养老家政、文化娱乐、旅游观光等消费服务，鼓励汽车、家电、住房装修等大宗消费，加快培育新的消费增长点。加强城乡流通基础设施和市场体系建设，在中心城市构建购物消费15分钟城市生活圈，在每个县建设或改造1个农贸市场，完成农村便民连锁店信息化改造500个。大力推进“农超对接”、“农校对接”、“农早对接”、“农批对接”，畅通农产品进城、工业品下乡渠道，深入整顿和规范市场秩序，让群众放心消费、满意消费。

　　加快民营经济发展。全面落实支持政策，优化民营经济发展环境。引导民营企业加快建立现代企业制度，鼓励民营企业加强管理团队、营销团队、研发团队建设，加大对民营企业产品开发、品牌创建、市场开拓的支持力度，尽快培养一批标杆民营企业和优秀民营企业家。全年新发展1000个中小微型企业、新上100个转型发展项目、新增10个营业收入超亿元的“小巨人”企业，民营经济增加值、营业收入、利润总额、上交税金等主要指标力争增长16%以上。鼓励资源型企业按照“一矿一厂、一企一业”的要求，至少新上1个新型产业项目或非资源型产品项目，引导和激励民营经济转型发展。

　　着力服务企业。牢固确立“产业第一”的观念，把保增长的立足点放在保企业上，理直气壮地主动服务企业，切实帮助企业解决存在的问题，积极支持企业做大做强。认真落实各项税收优惠政策和收费减免政策，坚决杜绝各种不合理收费、变相收费、要求赞助和影响企业生产经营的参观检查等行为，切实减轻企业负担，营造良好经营环境。积极、主动、有效推动银企合作，多途径多形式破解企业融资难题，全年力争“助保贷”资金突破5亿元，全市优质企业融资需求获得银行更多资金支持，企业上市工作取得实质性进展。充分发挥行政审批服务中心的作用，不断改进工作作风，缩短办理时间，减少审批环节，提高审批效率。开展“带着公章下基层”活动，主动为企业办事提供服务。认真落实领导帮扶重点企业制度，及时帮助企业解决资金、技术、人才、用地、用电、用水、用气等方面存在的问题。

　　（二）加快转变经济发展方式，提高经济增长的质量。

　　改造提升传统产业。巩固发展煤炭资源整合、煤矿兼并重组成果，在确保安全的基础上，加快办理基本建设矿井手续，加快复工复产步伐，力争全市6座未开工矿井全部开工，力争神达望田煤业等10座以上矿井竣工投产，新增产能1200万吨以上，全年原煤产量突破6000万吨。拓展煤炭横向产业链，加快煤炭运销项目建设，确保偏关泰鑫煤业等5个洗煤厂投产，新增入洗原煤能力1000万吨以上；加强同铁路、公路部门的联系，进一步提升煤炭发运能力。加快铁矿企业各种手续的办理进度，在确保安全的基础上尽快组织生产；延伸铁精粉产业链条，实质性推进代县200万吨钢、繁峙县100万吨钢等两个钢铁项目的建设进度。大力发展火电、风电，确保河曲电厂二期2×660MW等新投产项目正常运行，确保华能神池太平庄等5个共计400MW风电项目、忻州洁晋2×12MW垃圾发电项目建成投产，加快河曲神东2×350MW项目建设速度，确保忻州广宇二期2×350MW项目开工，力争再有几个电力项目获得核准。积极发展建材业，加快推进华润忻州水泥、晋兴奥隆水泥项目建设，确保山西泰达、代县宏威两条日产4500吨新型干法水泥生产线和代县明利尾矿砂加气混凝土砌块制砖项目投产。加快推进忻州长城钨钼、金宇煅烧高岭土等项目。

　　培育壮大新型产业。切实加快中电投山西铝业三期、保德同德氧化铝项目建设进度。大力推进保德王家岭煤化工项目、宁武煤制烯烃项目等现代煤化工项目。加快装备制造业发展，确保原平佳诚液压、兴胜机械、高龙电力、天兰锅炉及定襄安宝锻压等项目投产；突出抓好阳煤忻通刮板机、美新液压支架等煤机装备业项目，在忻府、宁武、原平上马配套机修项目，建设采煤设备产业体系，在更好服务煤炭生产和煤炭运销中做好煤机装备制造这篇文章。加大旅游目的地和旅游集散地建设力度，着力提升旅游服务水平。按照建设“五个五台山”的要求，加快五台山提升改造，打造一流的旅游目的地；全力以赴抓好芦芽山国家5A级景区创建工作；以发展温泉水疗、温泉体验、温泉商贸、温泉地产为基础，加快温泉旅游集散地建设步伐，实现奇村、顿村、大营、汤头温泉游的互动；加强雁门关、河边、老牛湾等景区建设，打造一批新的旅游景点。加快发展商贸、物流业，重点推进忻府区东方家园物流中心、原平德金农副产品物流园区、原平豪德等一批物流项目；支持定襄法兰产业做大做强，建设集现代物流、出口监管仓库为一体的现代物流园区，打造生产服务业基地。紧紧抓住城镇化的历史机遇，抓紧布局产业项目，推动地产开发，通过城镇化聚集人流、物流、资金流、信息流，实现产业发展和城镇化的融合，加快推动城镇经济发展。

　　强化创新支撑。加快科技创新。引导和支持企业自主或联合建立科技研发中心。新评定3个以上省级技术中心，新认定2个以上高新技术企业，鼓励管家营等省级企业技术中心上档升级，实现全市国家级企业技术中心零突破。打造优质品牌。力争新认定山西省著名商标20个以上、山西省名牌产品6个以上，不断增强我市产品的市场竞争力。加快商业模式创新。鼓励探索新的发展领域，发现新的重要商机，尽快构建切合忻州实际的独特商业模式。推进金融创新。积极培育各类新型金融机构，建立完善金融服务平台，开发针对性和操作性较强的金融产品，发挥资本市场作用，提高企业直接融资比重，着力破解企业融资难题。提升金融服务水平，不断优化金融生态环境，有效防范金融风险。

　　抓好“一园六企”。认真落实县（市、区）长联系“一园六企”制度，确保“六企”项目见到实效。重点抓好忻州煤化工循环经济园区等10个市级工业园区和忻府区豆罗建材工业园区等20个县级工业园区建设，实现园区建设的重大突破。加大淘汰落后产能力度。今年再淘汰冶金行业1.12万吨落后产能、电力行业2台0.6万千瓦小机组、水泥行业40万吨落后产能。通过淘汰落后产能促进产业结构的转型升级。

　　加强经济运行监测。完善经济运行监测联席会议制度，及时分析研判经济走势。建立工业经济运行监测系统，做好煤、电、运等综合协调，强化要素保障，及时解决经济运行中的突出问题。

　　（三）做好“三农”工作，推动农业农村大发展。

　　大力推进农业产业化。认真落实各项补贴政策，充分发挥杂粮生产优势。全市杂粮面积保持在230万亩以上，粮食总产稳定在25亿斤以上。努力提高设施农业效益。新发展温室大棚1万亩，完善新建千亩园区5个、百亩园区50个、50亩园区50个，充分发挥农业设施园区的示范带动作用。新发展“一村一品”示范村270个，加快8个省级“一县一业”县建设，启动神池、五寨、岢岚等养羊重点县人均5只羊建设规划，全市新发展羊60万只。加快发展标杆性龙头企业。新增销售收入百万元以上的龙头企业不少于10个，农产品加工龙头企业销售收入达到46亿元以上。加快品牌打造，新认证“三品”不少于50个。加强农畜产品质量安全监管，强化动物疫病防控，确保不发生大的疫情。

　　切实加快扶贫开发。坚持扶贫跟着产业走。把产业扶贫作为扶贫开发的核心，启动静乐县片区开发和“两山”特困连片地区扶贫开发项目，整村推进100个村。通过培育产业、项目带动，形成产业化扶贫开发的新局面。坚持搬迁跟着意愿走。把易地扶贫移民搬迁作为扶贫开发的有力举措，全年搬迁安置4万人，减贫9万人，贫困县农民人均纯收入突破5000元。加强移民小区基础设施、公共服务、社会管理、群众教育等后续工作，真正使农民成为市民、移民小区成为文明小区。加大农民培训力度。培训农民13万人，新转移劳动力3.5万人，进一步提高农民的工资性收入，力争农民收入增幅继续超过城镇居民收入增幅。

　　培育农村新型经营主体。坚持和完善农村基本经营制度，认真落实农民的土地承包经营权。着力培育新型经营主体和多元服务主体，大力扶持专业大户、家庭农场。积极发展农民专业合作社。认真抓好400个示范社建设，不断探索多种形式的规模经营。着力完善“一乡一站”、“一村一点”科技服务全覆盖工程，力争全市168个乡镇或区域农技推广机构建设项目全部达标。

　　进一步发展农村公共事业。加快新农村连片建设，推进城乡规划、基础设施、产业发展、生态建设、公共事业“五个一体化”，有效改善农村公共服务。认真落实“十个全覆盖”工程管理办法，巩固提升建设成果。扎实办好农村困难家庭危房改造、特困群众易地搬迁、行政村街道亮化、村级幼儿园改扩建和乡村清洁工程五件实事。不断深化农村低保、医保、养老保险等各项措施，切实做到公平公正、群众服气。

　　（四）加快市域城镇化进程，提高城镇化发展质量。

　　坚决维护规划权威。强化规划引领，实现规律主导，坚持按照规划进行建设。加快编制忻州城区控制性详细规划、忻州老城保护规划以及交通、供水、供热、供气等专项规划。科学设置前置条件，强化“一书两证”管理，健全科学有效的工作制度，形成环环相扣、互相监督的良好机制。

　　大力实施城区重点城建工程。在继续完成“7451”后续工程、全面建成“五馆一院”和忻顿路桥的基础上，以拓宽出入口、建成东环路、贯通中轴线、改造旧街巷、畅通微循环、美化新城区、建设特色街为重点，大力实施第二个“大干城建年”重点工程，进一步提升承载能力，改善人居环境，提高城市品位，为打造百万人口市域中心城市奠定坚实基础。完成广宇煤电150万平方米集中供热项目，新增绿地面积200万平方米，抓好新建道路管网配套、城区老旧管网改造和天然气置换人工煤气工程，加强城市供水体系建设，抓好张家庄村等3个城中村改造试点工作。推动各县（市）开工一批新的城建重点工程，实施好7个“百镇建设”示范镇工程，加快扩容提质的步伐。

　　依法整顿土地和建筑市场。巩固忻州市区防范和查处违法建设成果，严格土地用途管理，把整顿工作不断引向深入。大力支持依法建设，切实提高服务水平，为群众建设创造条件。从严防范和查处违法建设，落实举报违建重奖制度，发挥媒体及全社会的监督作用。支持各县（市）加大土地、建筑市场秩序整顿力度，进一步形成依法建设、拒绝违建的良好风气。

　　不断提高城市管理水平。发挥市民的主人翁作用，营造人民城市人民建、人民城市人民爱的浓厚氛围。发挥忻州城区数字化城管平台作用，提高城市科技监管水平。加快城区市容环境基础设施和便民市场建设。制定专项管理制度，开展以“治脏、治乱、治差”为主题的城市综合整治活动，改善城区环境卫生和交通秩序。加强城市公交管理，提升公交运营水平和服务质量。

　　有序推进农业转移人口市民化。认真落实政策规定，加快推进户籍制度、社会管理相关制度改革，健全城市服务体系，逐步实现城镇基本公共服务覆盖常住人口，为人们自由迁徙、安居乐业创造公平的制度环境。

　　（五）大力改善生态环境，加快建设美丽忻州。

　　抓好节能工作。继续坚持用电量指标与GDP挂钩，抓好重点行业、企业的节能工作。加大对列入省“千家企业”的61户企业和年能源消费量1000吨以上标煤的105户工业企业的节能监察力度。增强经信、统计、电力部门之间和市、县之间的联动，不断完善节能工作机制。抓好可再生能源建筑应用示范城市项目建设，新改造40万平方米。通过采取综合措施，确保完成年度节能目标。

　　抓好减排治污。继续抓好火电、水泥等行业烟气脱硫、脱硝和城镇生活污水处理，实质性推进农业源减排，确保完成年度减排任务。抓好大气污染防治，完善大气环境质量预警预报制度，加大城区环境噪声控制工作力度。强化重点流域水污染防治，加大汾河、滹沱河、南云中河、恢河治理力度，确保各个监测断面水质达标。扎实开展市区创建国家环保模范城申报工作，大力推进县（市）创建省级环保模范城市工作，力争再有1—2个县通过省创模领导组验收。

　　抓好造林绿化。完善林业发展总体布局，加强林业项目规划和林地管护。落实生态补偿政策，巩固扩大林改成果。扎实推进“山上治本”、“身边增绿”和“产业富民”，确保全市新增营造林60万亩，森林覆盖率提高1个百分点以上。

　　（六）切实保障和改善民生，提高人民群众物质文化生活水平。

　　积极扩大就业。统筹做好农民工、城镇就业困难人员和退役军人等群体的就业工作，重点做好高校毕业生就业工作。加强职业技能培训，完善就业援助制度，建立重点建设项目用工对接制度，落实好各项就业扶持政策，以创业带动就业，确保实现全年就业目标。加强劳动监察和争议调解仲裁，构建和谐劳动关系。认真落实保障农民工工资发放制度，确保不拖欠农民工工资。

　　办好人民满意的教育。新建14所标准化幼儿园，新建、改扩建30所幼儿园。启动忻府区等8个县（区）和市直12所学校的义务教育学校标准化建设，启动云中新区学校配套工程。积极支持职业教育、特殊教育、继续教育，促进各级各类教育全面协调发展。提高教学质量，提升办学水平。下决心减轻小学生课业负担，坚决治理乱补课现象，下功夫解决城镇个别学校大班额、个别教师占编不在岗等问题，让群众和家长看到教育工作出现的新变化。

　　提升城乡社保全覆盖工程。扩大社会保险覆盖面。提高事业单位人员补助标准，稳妥开展事业单位养老保险制度改革试点，加大农民工、非公经济组织和灵活就业人员参保扩面工作力度，破解特殊群体参保难题。提高企业退休人员基本养老金，提高城乡居民月均基础养老金、城乡低保月均补助标准以及农村集中供养、分散供养的五保对象供养标准。完善社会救助体系和被征地农民社会保障政策。推广社会保障“一卡通”。

　　提升群众健康水平。完善基本药物制度和基层医疗卫生机构运行新机制，全面推动县级公立医院综合改革。健全全民医保体系，建立重特大疾病保障和救助机制，开展基本医疗保险按病种付费试点工作。继续实施基本公共卫生服务项目，加强重大传染病防控。加快新建市人民医院步伐，完善市中医院医技康复楼配套设施，开工建设忻州市儿童医院，完工14个县级卫生监督所。积极推进原平市、五台县、保德县以及定襄县河边镇、五台县台怀镇、繁峙县砂河镇创建国家卫生城镇工作。坚持计划生育基本国策，落实计划生育家庭扶助政策。提高食品药品安全监管水平，坚决杜绝重大食品药品安全事故。

　　改善城乡居民居住条件。新开工建设城镇各类保障性住房17146套，完成农村危房改造年度任务。开展农村房屋防震加固改造试点工作。加大房地产市场监管，实现市县房地产市场信息资源共享。

　　努力满足人民群众的精神文化需求。践行社会主义核心价值观，引深精神文明创建活动，着力提升公民素质。加强互联网监管，推进文明上网、文明用网。加快县级“三馆一院”等文化基础设施建设。实施“送戏下乡”和“文化惠民”工程，开展好“周末大戏台”、“乡村大舞台”活动，办好第三届“梨花奖”舞台艺术大赛。发展体育事业，支持群众性体育活动，重视解决文体活动场所不足问题。

　　加强和创新社会管理。健全和完善重大决策社会稳定风险评估机制，畅通和规范群众诉求表达、利益协调、权益保障渠道，健全基层社会管理服务体系，创新流动人口和特殊人群管理。深入推进平安忻州创建，严厉打击各种违法犯罪活动。加强防灾减灾能力建设和应急管理，妥善应对各类突发事件。支持国防和军队建设，做好双拥、优抚安置和人防工作。发展妇女、儿童、老龄、残疾人和红十字会等事业。落实党的民族宗教政策。做好外事、侨务、对台等工作。

　　（七）狠抓安全生产，奠定坚实的安全基础。

　　认真落实安全生产两个主体责任。坚持把落实政府的安全监管责任放在首位。认真落实安全生产的综合监管、部门监管、行业监管、属地监管责任，做到安全生产监管全覆盖。严格履行安全生产属地管理责任，不论什么性质的企业，不论多高规格的单位，都要一律严格依法监管。通过落实政府的监管责任，确保落实企业的安全生产主体责任。狠抓各类企业的安全投入、安全培训、安全评定等工作，引深安全标准化创建，提高本质安全水平。

　　突出抓好重点行业的安全生产。深入开展专项整治活动，不断加强安全隐患排查治理，扎实开展打非治违活动。严厉打击任何名义、任何形式的私挖滥采行为，努力做到不揭一个盖、不毁一座山、不动一两煤。抓好八大行业安全生产，明确监管重点，防控事故发生。以防瓦斯、防水、机电管理为重点，坚决杜绝煤矿重特大事故发生。抓好交通运输行业事故防范，严格落实重要路段“双人包保”制度，严查各种违法行为，扭转交通事故高发势头。

　　切实加强安全生产基层基础工作。深入发动群众参与安全生产，推动安全文化进机关、进企业、进学校、进乡村、进社区、进家庭。继续实行隐患举报重奖制度，做到“有报必查、有患必改”。充分发挥安全档案作用，推进科技兴安和安全质量标准化建设。抓好企业基础管理，强化职工安全培训，从根本上提高安全保障能力。引导非煤矿山企业抓住二次整合机遇，在规模化、现代化、质量标准化上下功夫，实现安全健康发展。

　　严格安全生产责任追究。切实加强安全生产的组织领导，严格落实安全生产各项规定，建立健全安全生产长效机制。做好应急值守工作，及时发现和处置安全隐患，自觉接受群众、社会、媒体监督。严格安全执法、责任倒查和从严问责。严格安全生产工作考核，落实安全生产“一票否决制”，以铁的纪律推动全市安全生产形势向稳定好转坚实迈进。

　　（八）勇于探索创新，激发转型跨越发展的动力和活力。

　　认真抓好转型综改区建设。不断深化开放的广度和深度，切实建好“三个门户”、打造“三个集散地”、发展“六大经济板块”，培育八大产业。抓好五台山提升改造、沿黄流域生态修复等标杆项目。抓好土地利用、行政审批、人才引进等重点领域体制机制创新和改革。抓好“两县、两园、一板块”、“一县一企”、“一园六企”推进工作，力求在重点领域、关键环节和标杆项目建设上实现重大突破。大力支持原平、保德做好扩权强县试点工作。

　　稳妥推进国有困难企业改革。坚持依法办事、分类指导、分步实施、规范操作，坚定不移推进国有企业改革，千方百计帮助企业解困。今年下决心推进云河集团、云马焦化、煤机厂等企业的改革工作。

　　积极推进事业单位改革。完善事业单位人员竞聘上岗、绩效考核、业绩奖励等相关配套规定。完善岗位设置管理，建立“多劳多得、优绩优酬”的竞争机制。严格事业单位公开招聘制度，坚决防范事业单位进人上的不正之风。

　　不断深化行政审批制度改革。切实转变政府职能，推动政企分开、政资分开、政事分开、政社分开，确保行政机关按照法定权限和程序行使权力。进一步减少行政审批事项，真正向市场放权、向社会放权，发挥社会组织的作用，激发经济社会发展活力，从根本上遏制行政审批边减边增问题。严格事中事后监管，管住管好政府该管的事，提高政府管理科学化水平。

　　各位代表！今年我市经济社会发展的任务非常艰巨，对政府工作提出了新的更高要求。我们要进一步加强政府自身建设，严格落实对政府系统工作人员的“五章约法”，坚持不懈地推进法治政府、为民政府、服务政府、效能政府、诚信政府、廉洁政府建设，带头落实中央关于改进工作作风、密切联系群众的8项规定和省委、市委的贯彻意见，以崭新的工作作风、精神面貌和工作业绩取信于民。

　　强化宗旨意识。继续开展“给人民汇报、向人民问计、请人民评议”活动，多干群众满意的好事实事，多解决一些事关群众切实利益的问题。坚持勤俭办事。牢固树立过紧日子的理念，不折不扣地落实国务院关于政府性楼堂馆所一律不得新建、财政供养人员只减不增、“三公”经费只减不增的“约法三章”。恪守工作职责。“在其位，谋其政”。干一事，成一事。不怨天尤人，不推诿扯皮。守卫法律底线。坚持依法办事，一切在法律政策范围内活动。坚持运用法治思维和法治手段解决问题、推动工作。保持敬畏心理。敬畏群众、敬畏权力、敬畏责任。遇到问题多从自身找原因，以一日三省的态度对待工作。规范工作秩序。讲规矩，讲原则，讲程序。严格各项纪律。坚决执行政治纪律、工作纪律，全面落实党风廉政建设各项规定。发挥表率作用。要求群众做到的政府首先做到，要求群众不做的政府坚决不做。以身作则，身体力行，努力创造上下同心、群策群力、干事创业的良好局面。

　　各位代表！转型跨越使命光荣，全面小康前景灿烂。我们一定要在市委的坚强领导下，认真实施“3581”发展战略，扎实苦干，攻坚克难，赶队前行，进位争先，努力完成各项任务，为把忻州建设成为富裕文明、开放和谐、充满活力的新型工业旅游城市而奋斗！
